# Correction: ORFanID: A web-based search engine for the discovery and identification of orphan and taxonomically restricted genes

**DOI:** 10.1371/journal.pone.0308834

**Published:** 2024-08-08

**Authors:** Richard S. Gunasekera, Komal K. B. Raja, Suresh Hewapathirana, Emanuel Tundrea, Vinodh Gunasekera, Thushara Galbadage, Paul A. Nelson

There are errors in the Funding statement. The correct Funding statement is as follows: This work was funded partly by Discovery Institute, Seattle Washington, and the Roth family. The funders did not and will not have a role in study design, data collection and analysis, decision to publish, or preparation of the manuscript. [1]
