# Supplementary material for: ORFanID: A web-based search engine for the discovery and identification of orphan and taxonomically restricted genes
Source: PLoS One. 2023 Oct 25;18(10):e0291260. doi: 10.1371/journal.pone.0291260 (PMC10599687; doi:10.1371/journal.pone.0291260)
Supplement: S1 Table — (PDF) [file pone.0291260.s001.pdf]

**Supplemental Table 1.** Test results of ORFanID functionality.

| **Species** | **Taxonomy ID** | **Accession** | **ORFanID Classification** | **Comments & References** |
|---|---|---|---|---|
| Drosophila melanogaster | 7227 | NP_649722.1 | strict ORFan | Orphan gene based on flybase.org. Based on unpublished data, this protein has a role in eye development. |
| Drosophila melanogaster | 7227 | NP_727262.2 | strict ORFan | Orphan gene based on Levine et al., 2006. Flybase.org and ortho database show orthologs in Drosophila genus species. |
| Drosophila melanogaster | 7227 | NP_652406.1 | strict ORFan | Orphan gene based on Levine et al., 2006. No orthologs in orthodb. |
| Drosophila melanogaster | 7227 | NP_723266.1 | strict ORFan | Orphan gene based on Levine et al., 2006. No orthologs in orthodb. |
| Drosophila melanogaster | 7227 | NP_608405.4 | strict ORFan | *de novo* gene @ sub-group level based on Chen et al., 2007 & ortho db shows this protein in Drosophila sibling species, including *Drosophila melanogaster.* No orthologs in non-Drosophila insects or other organisms according to flybase.org |
| Drosophila melanogaster | 7227 | NP_726607.1 | Phylum | |
| Drosophila melanogaster | 7227 | NP_728356.2 | strict ORFan | Uncharacterized protein and no orthologs in flybase.org |
| Drosophila melanogaster | 7227 | NP_001097034.2 | strict ORFan | Reinhardt and Jones, 2013 |
| Drosophila melanogaster | 7227 | NP_001162900.1 | strict ORFan | Uncharacterized protein and no orthologs in flybase.org |

| | | | | |
|---|---|---|---|---|
| Drosophila melanogaster | 7227 | NP_001259080.1 | class | |
| | | | | |
| C.elegans | 6239 | NP_500848.2 | order | TRG based on Verster et al., 2017. WormBase & orthodb show Nematode orthologs |
| C. elegans | 6239 | NP_494931.1 | orfan gene | TRG based on Verster et al., 2017. WormBase & orthodb show orthologs in Caenorhabditis genus |
| | | | | |
| S.cervisiae | 4932 | NP_001335743.1 | strict ORFan | *de novo* gene. No orthologs in other databases |
| S. cervisiae | 4932 | NP_014130.1 | strict ORFan | Strict Orphan based on publication (Cai J et al., 2008).BSC4 may be involved in the DNA repair pathway during the stationary phase of S. cerevisiae and contribute to the robustness of S. cerevisiae, when shifted to a nutrient-poor environment. |
| S. cervisiae | 4932 | NP_015017.1 | strict ORFan | Essential TRG based on Verster et al., 2017 |
| S. cervisiae | 4932 | NP_010526.1 | strict ORFan | Essential TRG based on Verster et al., 2017 |
| S. cervisiae | 4932 | NP_011695.1 | strict ORFan | Essential TRG based on Verster et al., 2017 |
| S. cervisiae | 4932 | NP_011030.3 | strict ORFan | Essential TRG based on Verster et al., 2017 |
| S.cervisiae | 4932 | P47132.1 | strict ORFan | Strict Orphan based on publication. |
| S.cervisiae | 4932 | NP_009873.1 | strict ORFan | Saccharomyces specific gene based on few publications |
| | | | | |

| | | | | |
|---|---|---|---|---|
| H. sapiens | 9606 | NP_073152.2 | Genus | Order restricted based on a publication and family based on ortho database |
| H. sapiens | 9606 | NP_001316897.1 | order | Order restricted based on a publication. Orthodb shows conservation in eutheria clade |
| H. sapiens | 9606 | NP_872390.2 | order | It is a human-specific gene based on a publication. No conserved domains in NCBI database. Eutheria clade based on orthodb |
| H. sapiens | 9606 | Q1W209.2 | family | It is a human-specific gene based on a publication |
| | | | | |
| Danio rerio | 7955 | XP_002665008.4 | ORFan gene | It was shown to be genus restricted. According to orthodb, this protein has orthologs in actinopterygii class |
| Danio rerio | 7955 | NP_571379.1 | class | Should be present in all seeing animals. should be multi-domain |
