# Supplementary material for: ORFanID: A web-based search engine for the discovery and identification of orphan and taxonomically restricted genes
Source: PLoS One. 2023 Oct 25;18(10):e0291260. doi: 10.1371/journal.pone.0291260 (PMC10599687; doi:10.1371/journal.pone.0291260)
Supplement: S2 Table — (PDF) [file pone.0291260.s002.pdf]

Supplemental Table 2. abSENSE vs. ORFanID comparison.

Organism	NCBI Protein Accession Number	ORFanID Classifier	Result
Fungal	NP_015174.1	Class	1676102403557_GlL
Fungal	NP_116703.5	**Strict ORFan**	1676139596145_gQr
Fungal	NP_012059.3	**Strict ORFan**	1676139596145_gQr
Fungal	NP_011401.1	Genus	1676139596145_gQr
Fungal	NP_014025.1	Kingdom	1676139596145_gQr
Fungal	NP_010070.1	**Strict ORFan**	1676139596145_gQr
Fungal	NP_013577.1	Family	1676139596145_gQr
Fungal	NP_010143.1	Family	1676139596145_gQr
Fungal	NP_010972.1	Family	1676139596145_gQr
Fungal	NP_010486.3	Genus	1676141964287_UiA
Fungal	NP_012181.3	**Strict ORFan**	1676141964287_UiA
Fungal	NP_014964.1	Genus	1676141964287_UiA
Fungal	NP_009719.3	Family	1676141964287_UiA
Fungal	NP_010398.3	**Strict ORFan**	1676141964287_UiA
Fungal	NP_014522.1	Family	1676141964287_UiA
Fungal	NP_012054.1	**Strict ORFan**	1676141964287_UiA
Fungal	NP_010175.1	Phylum	1676141964287_UiA
Fungal	NP_010486.3	Genus	1676143078659_3qE
Fungal	NP_009757.1	Family	1676143078659_3qE
Fungal	NP_012587.3	**Strict ORFan**	1676143078659_3qE
Fungal	NP_015256.1	**Strict ORFan**	1676143078659_3qE
Fungal	NP_013688.1	Kingdom	1676143078659_3qE
Fungal	NP_010017.2	Genus	1676143078659_3qE
Fungal	NP_013113.1	Kingdom	1676143078659_3qE

Organism	NCBI Protein Accession Number	ORFanID Classifier	Result
Fungal	NP_015111.1	Genus	1676143078659_3qE
Fungal	NP_116686.3	Genus	1676144099201_5oG
Fungal	NP_010885.1	Genus	1676144099201_5oG
Fungal	NP_014908.1	Genus	1676144099201_5oG
Fungal	NP_012282.3	**Strict ORFan**	1676144099201_5oG
Fungal	NP_013614.1	Genus	1676144099201_5oG
Fungal	NP_013929.1	Family	1676144099201_5oG
Insect	NP_001285241.1	**Strict ORFan**	1676207776266_kNn
Insect	NP_001097257.1	**Strict ORFan**	1676207776266_kNn
Insect	NP_731242.1	**Strict ORFan**	1676207776266_kNn
Insect	NP_572277.2	Order	1676207776266_kNn
Insect	NP_001163736.1	**Strict ORFan**	1676207776266_kNn
Insect	NP_001260070.1	Family	1676207776266_kNn
Insect	NP_001163602.1	Phylum	1676207776266_kNn
Insect	NP_001247103.1	Phylum	1676210851491_1sk
Insect	NP_648151.2	Order	1676210851491_1sk
Insect	NP_650536.1	Family	1676210851491_1sk
Insect	NP_001188817.2	Order	1676210851491_1sk
Insect	NP_611851.1	Family	1676210851491_1sk
Insect	NP_001262507.1	Order	1676210851491_1sk
Insect	NP_001137829.1	Genus	1676210851491_1sk
Insect	NP_996411.2	Class	1676232140982_SDo
Insect	NP_650020.2	Family	1676232140982_SDo
Insect	NP_572410.2	**Strict ORFan**	1676232140982_SDo
Insect	NP_612110.1	**Strict ORFan**	1676232140982_SDo

Organism	NCBI Protein Accession Number	ORFanID Classifier	Result
Insect	NP_001097941.1	**Strict ORFan**	1676232140982_SDo
Insect	NP_788714.1	Family	1676232140982_SDo
Insect	NP_523663.1	Phylum	1676232140982_SDo
Insect	NP_651860.2	**Strict ORFan**	1676232140982_SDo
Insect	NP_001246258.1	Order	1676234237302_RAr
Insect	NP_001262397.1	Order	1676234237302_RAr
Insect	NP_001286126.1	Order	1676234237302_RAr
Insect	NP_570044.1	**Strict ORFan**	1676234237302_RAr
Insect	NP_728059.1	**Strict ORFan**	1676234237302_RAr
Insect	NP_001287054.1	Family	1676234237302_RAr
Insect	NP_649652.4	**Strict ORFan**	1676234237302_RAr
Insect	NP_001285134.1	Class	1676234434226_fi4
