# Supplementary material for: ORFanID: A web-based search engine for the discovery and identification of orphan and taxonomically restricted genes
Source: PLoS One. 2023 Oct 25;18(10):e0291260. doi: 10.1371/journal.pone.0291260 (PMC10599687; doi:10.1371/journal.pone.0291260)

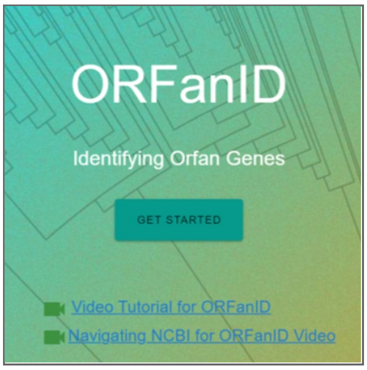

ORFanID Web-based Search Engine to Identify Orphan and Taxonomically Restricted Genes

Thushara Galbadage[1], Vinodh Gunasekera[2], Emanuel Tundrea[3], Richard S. Gunasekera[1]

[1]Biola University; [2]Chesalon USA, Inc.; [3]Emanuel University of Oradea

Thushara Galbadage
Biola University

ABSTRACT

ORFanID is a web-based software engine designed to identify ORFan genes from genomes of interest; from a given list of DNA or protein sequences within the NCBI databases. The selection of the taxonomy level of interest can define the scope of the search for orphan genes. Detectable homologous sequences are found by the software for candidate genes in the NCBI databases. Based on these findings, the ORFanID engine identifies and depicts orphan genes. Results may be viewed and analyzed graphically for scientific research and inquiry. As the enigma of orphan genes unravels, we believe ORFanID will provide critical insights into the origin, function, and prevalence of ORFan genes in genomes.

External link: http://www.orfangenes.com/

Protocol Info: Thushara Galbadage, Vinodh Gunasekera, Emanuel Tundrea, Richard S. Gunasekera . ORFanID Web-based Search Engine to Identify Orphan and Taxonomically Restricted Genes. **protocols.io** https://protocols.io/view/orfanid-web-based-search-engine-to-identify-orphan-cxubxnsn

Created: Jul 27, 2023

Last Modified: Aug 15, 2023

PROTOCOL integer ID: 85603

Keywords: ORFan, genes, ORFanID, NCBI, genomes

MATERIALS

ORFan Genes
Orphan genes, also known as taxonomically restricted genes, lack ancestral ties in other species at specific taxonomy levels. These genes present DNA and/or protein sequences lacking homology with those archived in prominent DNA databases like GenBank. Despite conventional beliefs attributing the emergence of new genes to processes like gene duplication or recombination, the widespread presence of orphan genes in sequenced genomes remains a mystery. This ubiquity represents a challenging question in the realm of life sciences.

Biological Implications
Traditionally, genes have been understood to dictate functions through proteins. Intriguingly, certain organisms, including *Hydra*, various mollusks, and salamanders, express unique proteins stemming from orphan genes. For instance:

• Hydra's anatomy is influenced by proteins produced by orphan genes.

• The mantles of specific mollusks owe their unique features to proteins resulting from orphan genes.

• The regenerative capability of salamander limbs can be attributed to proteins encoded by orphan genes.

Discovery through ORFanID
ORFanID stands as a powerful tool to unearth the origin, function, and broader implications of orphan genes. Capable of recognizing genes unique to diverse taxonomical levels such as genus, family, and species, ORFanID ensures precision by allowing adjustments in classification parameters. Thus, while some genes might be recognized as taxonomy-restricted based on set criteria, they might not strictly qualify as ORFans. This precision aids in pinpointing the sequence and functionality of de novo genes across various taxonomical spectra.

Accessing ORFanID

1 Navigate to ORFanID's webpage at http://www.orfangenes.com/

2 Click "Get Started" on the home page to access the search system.

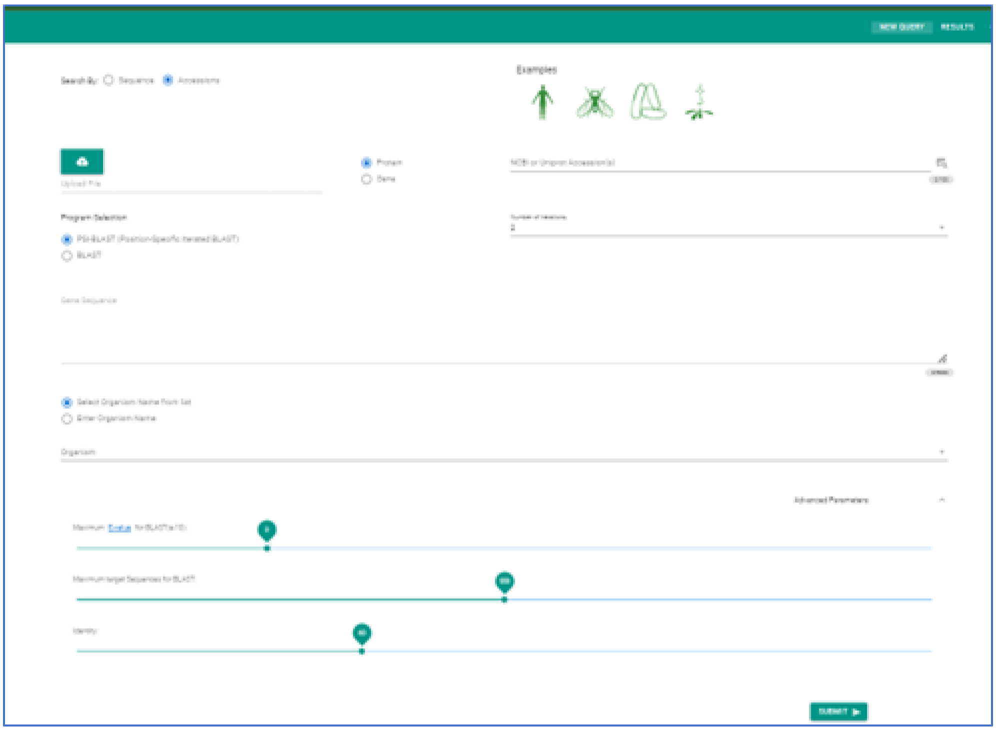

Sample Searches

3 If you don't have a specific gene sequence or an accession number, use the sample options provided. Four sample icons are located at the bottom left of the search screen. Clicking on any of these will pre-fill the input field with either a gene sequence or an accession number. Click "Search" to proceed.

Input Methods

4 Choose between searching for a gene sequence or using an accession number. For instance, to search a *Homo sapiens* sample, use the toggle switch to specify your preference.

5 ORFanID offers three search methods:
a. Uploading a FASTA file.
b. Inputting an accession number or numbers. (e.g., the *E. coli* sample has three given accession numbers)
c. Directly submitting gene sequences.

Search Guidelines

6 When searching multiple gene sequences, separate each with a new line or space.

7 You'll need to specify the organism for your search. If the desired organism isn't listed, refer to the NCBI taxonomy database to obtain its full scientific name and taxonomy ID. Enter this information, ensuring the taxonomy ID is in parentheses.

8 Choose between searching by protein or gene using the provided radio selection boxes.

Additional Options

9 There's an optional Nickname field on the upper left. Use it to label and identify your search results for future reference.

10 Adjust the three advanced parameters if needed. By default, the maximum e-value for the BLAST algorithm is set to three, and the maximum target sequences are set at 550. Hover over the pop-up for detailed info.

Submission and Results

11 After confirming your search criteria, the organism, and any other fields, click "Submit". A pop-up will prompt you to review your search. If satisfied, proceed to submit. Depending on the complexity of your query, results may take between 3 to 15 minutes.

12 You'll be redirected to the "Results" tab. A light green box indicates that processing is ongoing. Once complete, it turns dark green. To view your results, click the graph icon on the right of the results table.

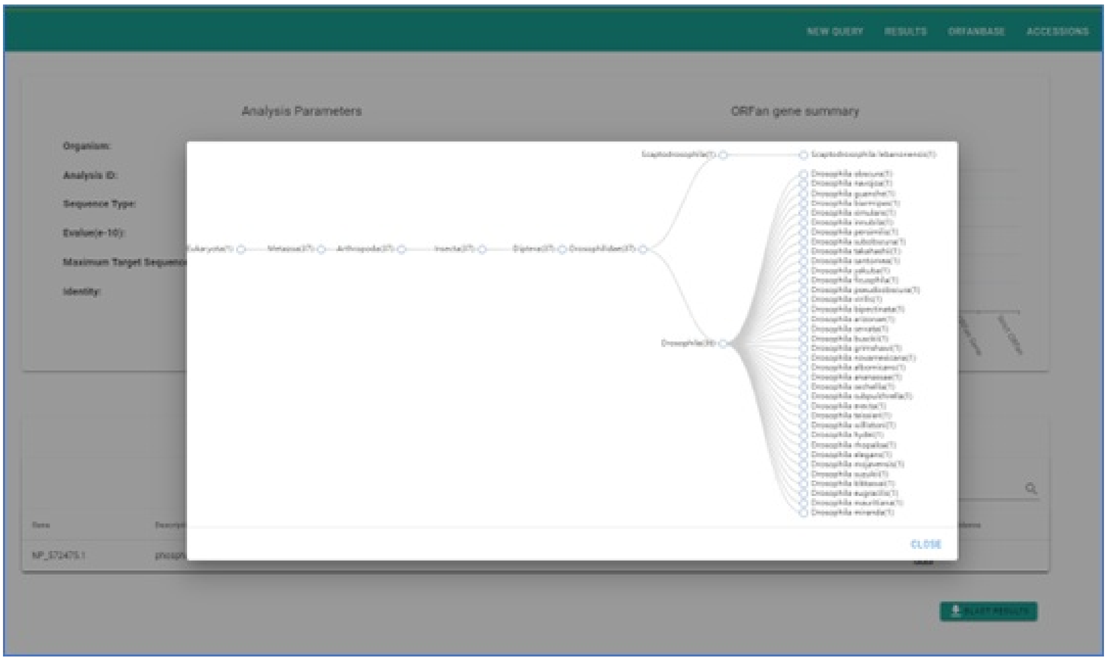

Supplement: S1 File — (PDF) [file pone.0291260.s003.pdf]
