# Supplementary material for: ORFanID: A web-based search engine for the discovery and identification of orphan and taxonomically restricted genes
Source: PLoS One. 2023 Oct 25;18(10):e0291260. doi: 10.1371/journal.pone.0291260 (PMC10599687; doi:10.1371/journal.pone.0291260)
Supplement: Attachment — Submitted filename: Response to Reviewers.pdf [file pone.0291260.s004.pdf]

Response to Reviewers: ORFanID: A Web-Based Search Engine for the Discovery and Identification of Orphan and Taxonomically Restricted Genes.

Academic Editor's Comments:

1. Item # 3: Acknowledgments Section of the manuscript:

   Funding information has been removed from the acknowledgement section.

   Amended statement for Online Submission Form: "This work was supported in part by the Roth Family. The funders did not and will not have a role in study design, data collection, and analysis, decision to publish, or preparation of the manuscript."
2. Item # 4 Regarding Table 1: "Table 1" is now mentioned correctly in the manuscript (in lines 247 and 250.)

Reviewer #1's Comments:

RE: 1. Why is there a minimum percent identity cutoff? I would like to see this either justified or removed.

We appreciate your insightful feedback concerning the use of a minimum percent identity cutoff in our homology search methodology, as presented in our paper on ORFanID.

Our decision to use a percent identity cutoff is an attempt to provide an additional filtering step that complements the E-value. While the E-value effectively reduces the number of false positives based on random matches, the percent identity cutoff can offer a more biologically meaningful filter. It is particularly beneficial when dealing with large protein families, where distant homologs may share low sequence identity but still have meaningful functional or structural conservation.

We chose a relatively high default percent identity cutoff of 60% to prioritize high-confidence homologs in our initial work with ORFanID. We acknowledge that many homologs can indeed share lower sequence identity, and the current cutoff could be restrictive in such cases.

Based on your valuable comment, we propose two potential modifications to address this concern. Firstly, we will lower the default percent identity cutoff value while still allowing users to increase it if they desire a more stringent filter. Secondly, we will provide clear guidelines in our ORFanID documentation about how and when to adjust the percent identity cutoff based on the user's specific requirements and the nature of their homology search.

We hope this adequately addresses your concern. Thank you for prompting us to clarify and refine this aspect of our software.

RE 2: 2. For the sake of reproducibility, it's important to add a note somewhere in the saved or downloaded results regarding what version of the database(s) were queried.

Thank you for your valuable suggestion. We appreciate the emphasis on reproducibility and have incorporated it into our manuscript. The version and date of the database queried have been specified in line 246 of the revised manuscript. This information is provided in the opening sentence of the "3. Results and Evaluations" section.

We appreciate your insights on the optional practical issues. We consider these observations to be highly relevant and valuable. Therefore, we are committed to addressing these in the forthcoming version of ORFanID.

Reviewer #2's Comments:

Thank you for your detailed feedback, Reviewer #2. We greatly appreciate your comments and suggestions. Below are our responses to your concerns:

1. We agree with your suggestion to use the definition of orphan genes as those lacking detectable homology rather than absolute homology. This indeed better reflects the functionality of our program. We have revised the definition in line 53.

2. We are grateful for your advice about maintaining neutrality with respect to the various mechanisms that may produce orphan genes and TRGs. This is particularly pertinent as our manuscript presents a software application in the style of a "lab protocol". In response to your feedback, we have made revisions to the introduction. We now not only state that mechanisms such as divergence beyond recognition, de novo gene birth, and horizontal gene transfer can lead to the appearance of genes without detectable homologs outside a certain taxon but also clarify that our program, ORFanID, does not differentiate among these processes.

   We have informed users that it's their responsibility to interpret the plausible underlying mechanism for the identified orphan/TRGs, based on their specific case and supporting evidence. Hence, while the program identifies potential orphan/TRGs, the decision regarding the most likely contributing mechanism lies with the users, taking into account the existing possibilities and the context of their research. We believe this update will make our program more useful and its limitations clearer to its users.

3. We have revised the sentence on lines 74-79 for greater clarity. We apologize for any confusion, and we believe that the modified text will be more comprehensible. Below is the modified version.

   "The prevailing theory, given the monopoly of life found on Earth, posits that every gene in existence today descended from genes present in the Last Universal Common Ancestor (LUCA). This implies that the genes we see today are directly descended from sequences found in LUCA. Various molecular mechanisms: for example, gene duplication,

recombination, and divergence have given rise to novel protein-coding regions (or open reading frames) in the genome, and their associated cell functions [11-13].

More significantly, these origination processes are not expected to eliminate historical traces of their origins, but rather to continue to show these homologies according to prevailing theory. Essentially, this means that we should see a connection between the core set of sequences seen in LUCA and all the genes stemming from this early ancestor."

4. Your point about the use of TBLASTN is well taken. We did not initially include TBLASTN because of its computational intensity, but in light of your feedback, we have decided to incorporate it as an option in the forthcoming version of the program to account for variability in genome annotations across species.
5. We acknowledge the issue of contamination in databases and agree that it may affect the results. As a precautionary measure, we have added this as a study limitation to the discussion section of the manuscript (Lines 324 to 327) alerting users about potential contamination issues, although our program cannot directly control this concern, the NCBI databases have quality checks, manual Curation, and User Feedback redundancy to mitigate the effects of contamination.
6. We concur that the threshold for identifying non-orphans could be adjusted for more accuracy, especially in comparative studies. We have noted this in the manuscript (Lines 216 to 219), informing users about the importance of choosing an appropriate threshold.
7. We have reconsidered the suggested application of our tool for species identification. Upon reflection, we agree that widespread genes might be sufficiently species-specific to not necessitate the use of orphan genes for this purpose. Consequently, we have revised the text on lines 307-314.

Once again, we are grateful for your thorough review, which has significantly helped improve our manuscript's quality. We hope that our responses and revisions adequately address your concerns.
